# Reply to comment on Cruts et al. (2008), "Exposure to diesel exhaust induces changes in EEG in human volunteers" by Valberg et al

**DOI:** 10.1186/1743-8977-5-11

**Published:** 2008-08-02

**Authors:** Björn Crüts, Anique Driessen, Ludo van Etten, Håkan Törnqvist, Anders Blomberg, Thomas Sandström, Nicholas L Mills, Paul JA Borm

**Affiliations:** 1 Centre of Expertise in Life Sciences, Zuyd University, Heerlen, The Netherlands; 2 Department of Respiratory Medicine and Allergy, University of Umeå, Sweden; 3 Centre for Cardiovascular Sciences, The University of Edinburgh, UK

## 

We thank Valberg and colleagues for their critical reflections on our work. The comments mainly focus on the relevance of our original findings to common ambient exposure to diesel exhaust fumes and particulate matter (PM) as well as the mechanism of the observed changes in EEG. In our manuscript [1] we described changes in quantitative EEG activity at frequencies above 20 Hz following exposure to diluted diesel engine exhaust. We do not provide mechanistic insight into how this exposures influences cerebral function and therefore interpretation of our observation is entirely based on hypothesis. We did not, as Valberg and colleagues [2] suggest, make any definitive conclusions regarding the effects of ambient particles on the development of neurological disease, but rather suggest that further research is required to explore these potentially important observations.

We are sorry if, in reading our paper, Valberg and colleagues [2] felt that we have been inappropriately definitive in our conclusions. By highlighting the following extract from our discussion, "our findings are due to an effect of nanoparticles that slowly penetrate the brain or affect neurophysiologic signaling", the authors do not acknowledge the carefully considered discussion that we put forward in the manuscript. This extract placed in context reads as follows: "Based on the onset, location, frequency profile and persistence of the response in EEG, we suggest that our findings are due to an effect of nanoparticles that slowly penetrate the brain or affect neurophysiologic signaling. However we can only speculate what these effects on the frontal cortex may mean for chronic exposure to PM and/or nanoparticles." Moreover, in the preceding text we concentrate on potential interference of gaseous compounds in the diesel exhaust. Since the study reported by Cruts et al [1] we have made significant progress and have performed similar studies with volunteers exposed to (1) lower concentrations of diesel with less contaminant gases, and (2) spark-generated carbon nanoparticles at different concentrations to evaluate whether the effect is driven by nanoparticles. We hope to report soon on the outcome of these studies in this or other journals.

Many of the comments on the validity of our findings are based on assumptions that EEG is sensitive to normal conditions, such as diurnal variation, exercise and intake of nutritional components with pharmacological activity such as ethanol, nicotine, caffeine and others. It needs little discussion that we are aware of those effects and have made rigorous attempts to control for these factors. Intake of alcohol, drugs used for neurological diseases, and sleep deprivation were exclusion criteria in the study. Moreover, each subject was his own control and exposed at exactly the same time of day to either sham or diesel exhaust. The different exposures were separated by 2 days to limit possible extended effects of the diesel exposure. In addition, all parameters were calculated as changes over exposure and post-exposure period, and baseline parameters at the start of sham and diesel exposure were not significantly different. Exercise and concentration were controlled by a similar cognitive task (reading) without exercise at all, in contrast to earlier studies using the same unit. Perhaps most important is the fact that the changes that we observed in EEG are in frequency domains (β2) that were not studied at all in the paper that is referred by Valberg and colleagues in their letter [3]. In fact the latter paper reports circadian rhythms for theta, alpha and β1-frequency bands only. The β2-activity (20–32 Hz) which was found to be increased in our study was not investigated in relation to the circadian rhythm.

It is interesting to note that Cummings et al [3] use the same measurement period and design (3 min eyes open, 3 min eyes shut) as we used to control our baseline, and which was used to demonstrate the changes as shown in Table 1. In addition we performed a continuous measurement of EEG over a 2 hr period and the data depicted in the graphs are a result of that analysis. Therefore the power of our design and (statistical) analysis of changes over time is superior to the 3 min interval evaluations.

Finally, the authors suggest that our study is limited as the exposure is not representative of current New Technology Diesel Exhaust (NTDE) emissions. NTDE is a term used only by the authors and first reported in the literature as recently as 2006. The overwhelming majority of diesel-powered vehicles in use today are not powered by Green Diesel Technology^® ^as developed by the author's employers, the International Truck and Engine Corporation. The aim of our research program is to determine the health effects of exposure to air pollutants with a view to informing legislation for the use of new technologies and public health interventions to protect the public. We are currently conducting studies assessing the potential benefits of diesel engine exhaust filters, and these studies on the effectiveness of new technologies need to be put into context, and comparisons drawn with existing technology. To suggest that it is not relevant to determine the health effects of emissions from older diesel engines when older technologies are the principle source of diesel emissions worldwide is misguided.

In the real ambient exposure study by McCreanor et al [4] average levels of PM2.5 and PM10 were 28 and 125 μg/m^3 ^respectively. This study was conducted in the centre of London, one of the first cities to introduce congestion charging to reduce exposure to air pollution and protect the health of its inhabitants. Unfortunately, these traffic restrictions are not in place in the majority of the world's cities or workplaces where PM concentrations regularly reach levels of 300 μg/m^3^. A major proportion of this local PM mass is attributable to combustion-derived nanoparticles from traffic; ranging from 20% at city background remote monitoring sites up to 70% in a road tunnel. Exposure to 300 μg/m^3 ^during one hour increases a person's average exposure over a 24-hour period by only 12 μg/m^3^. Changes of this magnitude occur on a daily basis even in the least polluted cities, and are associated with increases in morbidity. Furthermore, the gaseous co-pollutants in our exposure are all well within the levels recommended by the US Occupational Safety & Health Administration for employees working with diesel exhaust. Therefore, we strongly believe that our model is highly relevant both in the composition and magnitude of exposure for the assessment of short-term health effects in man. Last but not least, even if this exposure would not be environmentally relevant, it would be a new effect at relatively short peak exposures that does easily occur. We agree with Valberg et al. that there is a need to explore the significance of this finding in a broader context, such as chronic exposure to lower levels of diesel exhaust fumes.

In summary the reported study [1] was conducted in such a way that described artefacts are highly unlikely to have played a role in these effects. In addition, the report was only submitted when we repeated the study in an independent setting at lower concentrations where preliminary findings suggest a similar effect. We therefore feel confident that these findings merit the attention of not only readers of "Particle and Fibre Toxicology", but also of public health professionals and researchers that deal with the complex issue of air pollution. We thank Valberg and colleagues for raising this issue in their letter [2], since it gives us the opportunity to detail our work and announce follow-up publications.

## Competing interests

The authors declare that they have no competing interests.

## Authors' contributions

All authors read and approved the final manuscript.
